# Emerging Biomedical and Clinical Applications of 3D-Printed Poly(Lactic Acid)-Based Devices and Delivery Systems

**DOI:** 10.3390/bioengineering11070705

**Published:** 2024-07-11

**Authors:** Allan John R. Barcena, Prashanth Ravi, Suprateek Kundu, Karthik Tappa

**Affiliations:** 1 Department of Interventional Radiology, The University of Texas MD Anderson Cancer Center, Houston, TX 77030, USA; ajbarcena@mdanderson.org; 2 College of Medicine, University of the Philippines Manila, Manila 1000, Philippines; 3 Department of Radiology, University of Cincinnati, Cincinnati, OH 45219, USA; raviph@ucmail.uc.edu; 4 Department of Biostatistics, Division of Basic Science Research, The University of Texas MD Anderson Cancer Center, Houston, TX 77030, USA; skundu2@mdanderson.org; 5 Department of Breast Imaging, Division of Diagnostic Imaging, The University of Texas MD Anderson Cancer Center, Houston, TX 77030, USA

**Keywords:** 3D printing, drug delivery, medical device, polylactic acid, surgery, tissue engineering, radiotherapy, regenerative medicine

## Abstract

Poly(lactic acid) (PLA) is widely used in the field of medicine due to its biocompatibility, versatility, and cost-effectiveness. Three-dimensional (3D) printing or the systematic deposition of PLA in layers has enabled the fabrication of customized scaffolds for various biomedical and clinical applications. In tissue engineering and regenerative medicine, 3D-printed PLA has been mostly used to generate bone tissue scaffolds, typically in combination with different polymers and ceramics. PLA’s versatility has also allowed the development of drug-eluting constructs for the controlled release of various agents, such as antibiotics, antivirals, anti-hypertensives, chemotherapeutics, hormones, and vitamins. Additionally, 3D-printed PLA has recently been used to develop diagnostic electrodes, prostheses, orthoses, surgical instruments, and radiotherapy devices. PLA has provided a cost-effective, accessible, and safer means of improving patient care through surgical and dosimetry guides, as well as enhancing medical education through training models and simulators. Overall, the widespread use of 3D-printed PLA in biomedical and clinical settings is expected to persistently stimulate biomedical innovation and revolutionize patient care and healthcare delivery.

## 1. Introduction

Poly(lactic acid) (PLA) is a versatile aliphatic polyester that can be synthesized from lactic acid monomers via direct polycondensation or ring-opening polymerization using a suitable catalyst [1]. Lactic acid, also known as 2-hydroxypropanoic acid, is one of the simplest hydroxy acids, and it contains one asymmetric carbon atom with two optically active configurations (L- and D-isomers). Hence, different stereochemical forms of PLA may be generated, which include poly(L-lactide), poly(D-lactide), and poly(DL-lactide). PLA was first synthesized in the 1930s by Wallace Carothers, a renowned American scientist credited with the development of neoprene and nylon at Dupont [2]. Since then, PLA has gained tremendous popularity in various fields due to its cost-effectiveness, versatility, and biocompatibility. It can be produced from starchy renewable sources including corn, wheat, and rice [3], making it more sustainable and less reliant on fossil fuels. Compared to petroleum-based polymer alternatives like acrylonitrile butadiene styrene (ABS) and nylon, PLA is also cheaper and simpler to produce [2]. Moreover, its thermoplastic nature allows for easy processing using various techniques, including three-dimensional (3D) printing. Indeed, the tunable nature of PLA’s physicochemical properties has been harnessed for the 3D printing of materials for various industrial applications, such as food packaging, textiles, automotive, and electronics [1,2]. In particular, its biocompatibility and sterilizability have made it an attractive material for biomedical and clinical applications [4,5,6]. PLA naturally undergoes degradation in situ via the process of hydrolysis, and its byproducts (i.e., lactic acid, water, and carbon dioxide) are easily metabolized by the body [7]. Hence, it can be used to fabricate medical devices that can safely interface with human tissues and organs. Overall, PLA’s affordability, combined with its versatility and biocompatibility, make it an ideal candidate for the fabrication of novel medical devices and delivery systems through 3D printing.

This review focuses on the wide range of the biomedical and clinical applications of 3D-printed PLA. While previous reviews have explored the general applications of 3D-printed PLA-based materials, this report consolidates the latest advancements in the 3D-printed PLA technology within the context of its transformative potential in patient care and healthcare delivery. This review aims to offer a comprehensive overview of the rapidly evolving field of 3D-printed PLA and its implications for the future of medicine by analyzing the diverse applications of this material in tissue engineering and regenerative medicine, drug delivery systems, medical devices, surgical instruments and guides, radiotherapy devices and phantoms, and training models.

## 2. PLA as a Versatile Material for 3D Printing

Three-dimensional printing, also referred to as additive manufacturing, is a revolutionary technology that enables the production of 3D objects from digital models [8,9]. This is accomplished by depositing successive layers of material until the entire object is formed, allowing the fabrication of more complex structures that would be difficult to produce using conventional manufacturing methods. Among the different materials used in 3D printing, thermoplastics, which become moldable above a specific temperature and return to a solid state upon cooling, have been widely used due to their ease of use and versatility. Specifically, thermoplastic PLA has become one of the most popular 3D printing feedstock materials because of its numerous advantageous qualities. As previously mentioned, PLA is biocompatible and is cheaper to produce than alternative petroleum-based polymers used in 3D printing. It has a glass transition temperature of around 55–60 °C, and it has a melting temperature of around 160–180 °C. In its capacity as a thermoplastic, it is capable of undergoing heating to its melting point, subsequent cooling, and re-heating without significant degradation [10]. It has good mechanical strength and may be combined with other substances to produce composites with novel characteristics. PLA filaments are also commercially available in a diverse array of colors and properties, providing users with a wide range of options and flexibility. This makes PLA a highly suitable material for use in a wide variety of 3D printing techniques. Several 3D printing methods, including binder jetting (BJT), material extrusion (MEX), material jetting (MJT), powder bed fusion (PBF), and vat photopolymerization (VP), have utilized PLA to produce biomedical and clinical constructs [1,2]. The standard terminologies and distinctions between these methodologies have been discussed in previous reviews [2,11].

**Figure 1:**
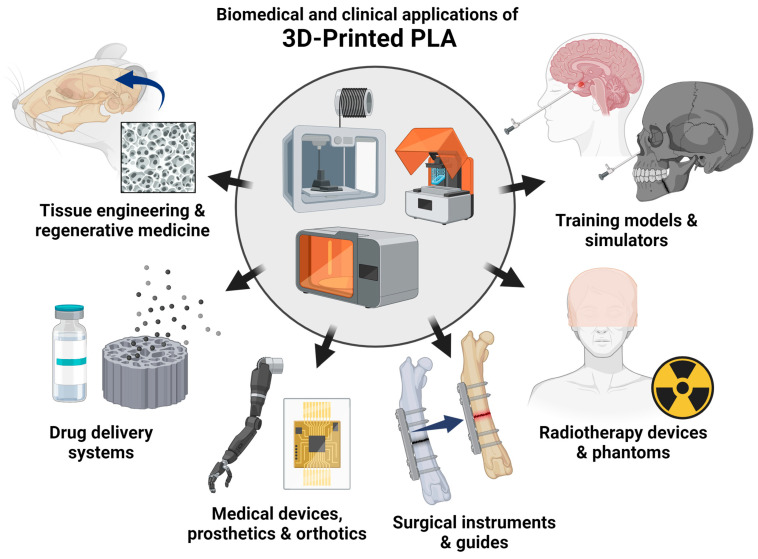
Emerging biomedical and clinical applications of 3D-printed PLA. The biomedical and clinical uses of PLA-based materials are discussed in the subsequent chapters: tissue engineering and regenerative medicine, drug delivery systems, medical devices, surgical instruments and guides, radiotherapy devices and phantoms, and training models (created using BioRender.com).

Ultimately, the 3D printing of PLA has enabled the fabrication of intricate scaffold geometries that can be customized for various biomedical and clinical purposes. The use of PLA-based materials in biomedical and clinical applications can be broadly classified into tissue engineering and regenerative medicine, drug delivery systems, medical devices, surgical instruments and guides, radiotherapy devices and phantoms, and training models (Figure 1).

## 3. Tissue Engineering and Regenerative Medicine

**Figure 2:**
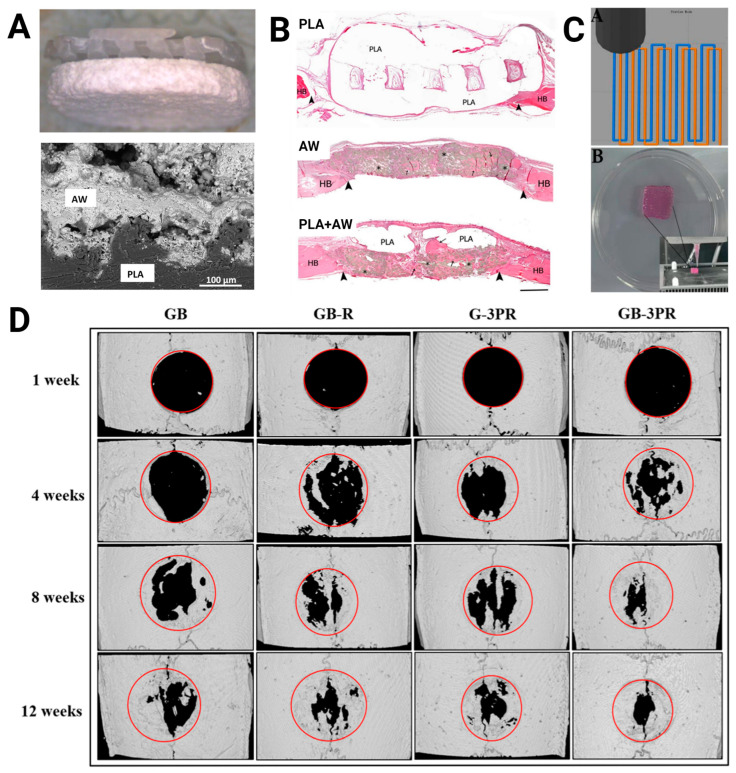
Three-dimensional-printed PLA in bone tissue engineering. (**A**) Composite disks composed of PLA and the bioactive ceramic apatite-wollastonite (AW) (top: photograph, bottom: scanning electron microscopy image of interface) showed (**B**) higher bone formation than either PLA or AW alone in rat calvarial defects after 12 weeks as seen via hematoxylin and eosin staining (bar = 1 mm; HB, host bone; arrows, new bone; arrowheads, defect margins; *, residual AW). Images were adapted with permission from Tcacencu et al. [21]. (**C**) Dual extrusion 3D-printed in situ vascularized scaffolds containing bone marrow-derived mesenchymal stem cells (MSCs) and rat aortic endothelial cells (RAOECs) or GB-3PR (top: path planning; bottom: printed scaffold) (**D**) exhibited higher bone formation than the other groups (i.e., single-nozzle 3D-printed gel with BMSCs [GB], single-nozzle 3D-printed gel with BMSCs and RAOECs [GB-R], and dual-nozzle 3D-printed gel with RAOECs [G-3PR]) in rat calvarial defects after 12 weeks as seen via computed tomography. Images were adapted with permission from Shen et al. [14].

Three-dimensional-printed PLA has gained significant attention in the domains of tissue engineering and regenerative medicine, specifically in the realm of bone tissue engineering, owing to its durability and adaptability. Nonetheless, PLA alone may not fully mimic the complex architecture and composition of natural bone, which is a dynamic structure composed of vascular canals, mineralized and unmineralized connective tissue matrix, and specialized cells. Hence, recent studies have blended PLA with different materials, such as polymers, ceramics, stem cells, and growth factors to enhance its physicochemical properties and functionality. The efficacy of 3D-printed PLA-based composite materials in bone tissue engineering has been demonstrated in several preclinical studies (Table 1, Figure 2). Both natural and synthetic materials have been combined with PLA to match the native bone’s biomechanical properties [12,13,14,15,16,17,18,19]. Bioactive ceramics have been added to PLA to enhance not only its mechanical properties but also its osteoconductivity or ability to promote bone growth [20]. Examples of ceramic materials that have been blended with PLA in the context of bone tissue engineering include apatite-wollastonite [21], beta-tricalcium phosphate [22], bioactive glass [15], and hydroxyapatite [23]. In addition, the incorporation of osteogenic cells and growth factors into the scaffolds has been shown to improve the regeneration of functional bone tissue. Several studies have shown that the addition of mesenchymal stem/stromal cells (MSCs) and extracellular vesicles (EVs), which have broad regenerative properties [24,25], can enhance the bone formation in rat calvarial defects [14,26,27,28]. Despite the recent numerous advances, several challenges need to be addressed to fully realize the potential of 3D-printed PLA-based materials in bone tissue engineering. These include replicating the intricate architecture of bone tissues, creating hierarchy and heterogeneity, and enhancing the vascularization. As previously mentioned, natural bone has a complex hierarchical structure that is difficult to reproduce using conventional techniques. Moreover, bone displays heterogeneity in structure and function across different locations. The development of high-resolution printing methods as well as strategies to improve oxygenation and nutrient transport, such as prevascularization and the incorporation of pro-angiogenic agents, can help overcome these challenges [29,30].

In addition to its use in bone tissue engineering, 3D-printed PLA has also been recently studied for nerve, skin, and vascular tissue regeneration [31,32,33,34,35]. In contrast to what is typically performed with bone scaffolds, PLA is combined with more pliable materials to mimic softer tissues. For example, to replicate the skin’s native architecture, PLA has been blended with softer natural polymers, such as hyaluronic acid and chitosan hydrogels [34,35]. Although the fabrication of soft tissues using PLA-based materials is still in its early stages, the functionality of these tissues is anticipated to be improved by future advancements in vascular tissue engineering and high-resolution printing. Overall, the aforementioned strategies highlight the adaptability of PLA in customizing scaffold characteristics to closely resemble the natural tissue environment and enhance the outcomes in tissue engineering and regenerative medicine.

**Table 1:** Preclinical in vivo studies on 3D-printed PLA scaffolds for bone tissue engineering.

Scaffold Components	Printing Technique	Model	In Vivo Results	Reference
PLA, apatite-wollastonite	MEX, BJT	Rat	After 12 weeks, the scaffold with apatite-wollastonite showed a higher amount of newly formed bone in the calvarial defects than plain PLA (BA: ~20% vs. ~2%).	[21]
PLA, HA, MSC	MEX	Rabbit	After 16 weeks, the scaffold loaded with cells demonstrated bone regeneration in the radial defects comparable to bone grafts (BV/TV: ~70%).	[23]
PLA, MSCs	MEX	Rat	After 12 weeks, both the cell-free and cell-seeded scaffolds exhibited bone regeneration in the calvarial defects (BA: ~55% vs. ~60%).	[27]
PLA, MSCs, EVs	MEX	Rat	After 6 weeks, the scaffold with MSCs and EVs showed the highest bone regeneration in the calvarial defects (BV/TV: 8.2%).	[26]
PLA, MSCs, EVs	MEX	Rat	After 6 weeks, the scaffolds with MSCs and engineered EVs had higher bone formation in the calvarial defects compared to plain PLA (BA: 9.72% vs. 0%).	[28]
PLA, nHA	MEX	Rabbit	After 12 weeks, the scaffold demonstrated new bone formation in the femoral defects (BV/TV: ~20%).	[36]
PLA, nHA, gelatin, PRP	MEX	Rat	After 12 weeks, the scaffolds with nHA, gelatin, and PRP promoted better bone regeneration in the calvarial defects than plain PLA (BA: 83.68% vs. 71.08%).	[12]
PLA, PDA	MEX	Rat	After 8 weeks, the etched scaffold with PDA promoted higher bone regeneration in the femoral defects compared to the untreated scaffold (BV/TV: ~30% vs. ~14%).	[13]
PLA, PEG, gelMA, MSCs, ECs	MEX	Rat	After 12 weeks, the scaffolds with MSCs and ECs exhibited the highest bone formation in the calvarial defects (BV/TV: ~33%).	[14]
PLA, PMMA, BG	MEX	Rabbit	After 4 weeks, the scaffolds with BG showed higher bone regeneration in the femoral defects compared to those without BG (BV/TV: 13–15% vs. 4–10%).	[15]
PLA, PTMC, HA	MEX	Rat	After 8 weeks, the PLA/PTMC/HA scaffold demonstrated lower bone regeneration in the femoral defects than the PTMC/HA scaffolds (BV/TV: ~13% vs. ~16%).	[16]
PLA, β-TCP	MEX	Rat	After 6 weeks, the scaffold with β-TCP showed significantly enhanced new bone formation in the femoral defects than plain PLA (BV/TV: ~38% vs. 25~).	[22]

Abbreviations: β-TCP, beta-tricalcium phosphate; BA, bone area; BG, bioactive glass; BJT, binder jetting; BV/TV, bone volume/total volume; EC, endothelial cell; EV, extracellular vesicles; gelMA, gelatin methacrylate; HA, hydroxyapatite; MEX, material extrusion; MSC, mesenchymal stem cell; nHA, nanohydroxyapatite; NP, nanoparticle; PDA, poly(dopamine); PLA, poly(lactic acid); PEG, poly(ethylene glycol); PMMA, poly(methyl methacrylate); PRP, platelet-rich plasma; PTMC, poly(trimethylene carbonate).

## 4. Drug Delivery Systems

**Figure 3:**
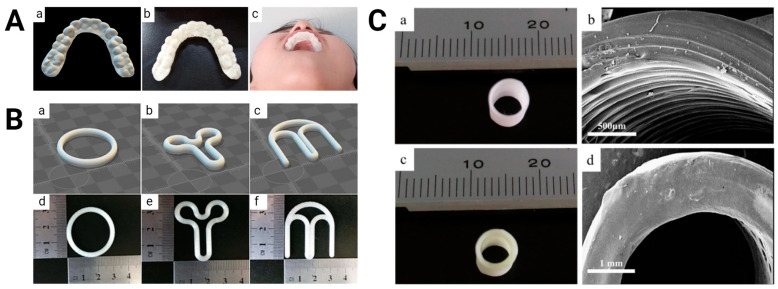
Three-dimensional-printed PLA-based drug delivery systems. (**A**) Computer-aided design image (left), printed version (middle), and worn example (right) of clonidine-loaded orthodontic retainer. Images were adapted with permission from Jiang et al. [49]. (**B**) Computer-aided design images (top) and printed versions (bottom) of progesterone-loaded O- (left), Y- (middle), and M-shaped (right) vaginal rings. Images were adapted with permission from Fu et al. [51]. (**C**) Printed versions (left) and scanning electron microscopy images (right) of bare (top) and nitric oxide-loaded (bottom) vascular grafts. Images were adapted with permission from Kabirian et al. [33].

PLA’s degradation kinetics can be controlled making it an excellent material for drug delivery. Three-dimensional-printed PLA has found use in drug-eluting bone scaffolds (Table 2), as well as medical devices and dosage forms (Table 3, Figure 3). By changing the composition of biodegradable scaffolds, the release rate of the encapsulated agents can be tailored to match the specific therapeutic needs, ensuring sustained and localized delivery with minimal toxicity. This capability is particularly valuable for chronic conditions or targeted therapies where precise dosage control is essential. In drug-eluting bone scaffolds, PLA matrices can be precisely engineered to deliver therapeutic agents such as growth factors, antibiotics, or anti-inflammatory drugs directly to the site of a bone defect, promoting tissue regeneration while preventing infection and inflammation. One of the most common agents loaded in bone scaffolds is bone morphogenetic protein 2 (BMP-2), an osteoinductive growth factor approved by the Food and Drug Administration (FDA) for human use [37]. Three-dimensional-printed PLA-based bone scaffolds loaded with BMP-2 have been shown to enhance the regeneration of bone in the calvarial defects in rodents [38,39,40,41], tibial defects in rabbits [42], and mandibular defects in pigs [43]. Other agents that have been delivered through 3D-printed PLA-containing bone scaffolds include amoxicillin [44], dexamethasone [45], ketorolac [44], and vancomycin [46,47].

Additionally, 3D-printed PLA-based medical devices, such as catheters [48], dental retainers [49], ocular inserts [50], vaginal rings [51], and vascular grafts [33], have been tailored to release a wide variety of agents, such as antibiotics, antivirals, anti-hypertensives, chemotherapeutics, and hormones, which could offer enhanced therapeutic efficacy and patient compliance. PLA’s versatility has also allowed for the fabrication of various dosage forms for the delivery of 5-fluorouracil [52], domperidone [53], and riboflavin [54]. Overall, the efficacy of 3D-printed PLA in drug elution is attributed to its capacity to accurately regulate the kinetics of drug release and its compatibility with an extensive array of therapeutic substances. Nonetheless, further studies are needed to optimize the composition of scaffolds and devices for maximizing the stability, safety, and efficacy of the incorporated drugs. 

**Table 2:** Preclinical in vivo studies on 3D-printed drug-releasing PLA scaffolds for bone regeneration.

Indication	Scaffold Components	Printing Technique	Model	Drug Release	In Vivo Results	Reference
Alveolar defect	PLA, PLGA, ketorolac, amoxicillin	MEX	Rat	The scaffolds released levels of ketorolac and amoxicillin above the minimum effective concentration for 17 and 30 days, respectively.	After 4 weeks, the drug-loaded scaffolds revealed less inflammation compared to the plain scaffolds.	[44]
Calvarial defect	PLA, alginate, collagen, BMP-2	MEX	Mouse	The scaffold demonstrated an initial burst release phase for BMP-2 on the first day (670.61 ng) followed by a gradual release phase up to day 14 in vitro (cumulative release: 1132.30 ng).	After 4 weeks, the scaffold treated with 2 µg/mL of BMP-2 showed the highest bone regeneration in the calvarial defects.	[38]
Calvarial defect	PLA, apatite, BMP-2	MEX	Rat	None reported	After 6 months, the scaffold with BMP-2 showed higher bone regeneration in the calvarial defects than the non-drug-releasing counterpart (BA: 44.85% vs. 31.2%).	[39]
Calvarial defect	PLA, Biogel, BMP-2	MEX	Rat	The scaffold demonstrated an initial burst release phase for BMP-2 in the first 4 days (85%) followed by a gradual release phase up to day 15 in vitro.	After 8 weeks, the drug-releasing scaffold showed higher bone regeneration in the calvarial defects compared to the plain scaffold (BV/TV: 23.41% vs. 2.94%).	[40]
Calvarial defect	PLA, PDA, alginate, heparin, BMP-2	MEX	Rat	The microspheres demonstrated an initial burst phase followed by a plateau for 2 weeks and a more rapid release. The microspheres released 58.1–59.1% of the incorporated BMP-2 during the first month.	After 12 weeks, the scaffold with BMP-2-loaded microspheres showed higher bone regeneration in the calvarial defects compared to the plain scaffold (16.7% vs. 4.6%).	[41]
Calvarial defect	PLA, PDA, PEI, pVEGF	MEX	Rat	The scaffold demonstrated a gradual release of pVEGF over 144 h (cumulative release: 15%).	After 4 weeks, the scaffold with pVEGF showed higher bone regeneration in the calvarial defects than the plain scaffold (~13% vs. ~8%).	[55]
Calvarial defect	PLA, PEG, nHA, dexamethasone	MEX	Rat	The scaffold demonstrated a cumulative release of 8.33% of loaded dexamethasone over 2 weeks.	After 4 weeks, there were no differences in the bone regeneration in the calvarial defects among the treatment groups.	[45]
Femoral defect	PLA, PLGA, nHA, vancomycin	MEX	Rabbit	The scaffold demonstrated an initial burst release phase for vancomycin on the first day (27%) followed by a gradual release phase up to day 24 in vitro (cumulative release: 62.3%).	After 4 weeks, there were no differences in the bone regeneration in the femoral defects between the drug-loaded and plain scaffolds.	[46]
Mandibular defect	PLA, BMP-2	MEX	Pig	None reported	After 3 months, the scaffold with 110 μg/cm^3^ BMP-2 demonstrated bone regeneration in the mandibular defects that are comparable to bone autografts (BA: ~20%).	[43]
Tibial defect	PLA, Biogel, MSC, BMP-2	MEX	Rabbit	None reported	After 4 weeks, the scaffold with BMP-2 and MSCs showed higher bone regeneration in the tibial defects than the plain scaffold (BV/TV: 7.9% vs. 4.31%).	[42]

Abbreviations: BA, bone area; BMP-2, bone morphogenetic protein 2; BV/TV, bone volume/total volume; HA, hydroxyapatite; MEX, material extrusion; MSC, mesenchymal stem cell; nHA, nanohydroxyapatite; NP, nanoparticle; PDA, polydopamine; PEI, polyethyleneimine; PLA, poly(lactic acid); PLGA, poly(lactic-co-glycolic acid); PEG, poly(ethylene glycol); pVEGF, vascular endothelial growth factor-encoding plasmid; SDF-1, stromal-derived factor 1.

**Table 3:** Preclinical studies on 3D-printed drug-releasing PLA-containing medical devices and dosage forms.

Device	Device Components	Printing Technique	Model	Drug Release	In Vivo Results	Reference
Catheter	PLA, gentamicin, methotrexate	MEX	In vitro	The catheter showed an initial burst release during the first few hours followed by a steady release for both gentamicin and methotrexate. The catheter also inhibited the growth of *E. coli*.	None reported	[48]
Dental retainer	PLA, PCL, PEG, clonidine	MEX	In vitro	The washed retainer released 0.42 mg of clonidine after 24 h followed by a stable release at a rate of about 0.24 mg per day over 3 days.	None reported	[49]
Implant	PLA, calcium carbonate, alginate, gemcitabine	MEX	Mouse	The implants demonstrated an initial burst release phase of gemcitabine for the first 10 h (24–29.3%) followed by a gradual release phase up to 3–7 days (cumulative release: 30–40%).	Over 4 weeks, the gemcitabine-loaded implants reduced the growth of subcutaneous MIA PaCa-2 tumors in mice.	[56]
Implant	PLA, methotrexate	MEX	Mouse	The implants demonstrated an initial burst release phase of methotrexate for the first day (25%) followed by a gradual release phase over 30 days (cumulative release: ~80%).	Over 3 weeks, the methotrexate-loaded implants significantly reduced the growth of subcutaneous 4T1 tumors in mice.	[57]
Implant	PLA, PCL, tetracycline	MEX	In vitro	The implants demonstrated a sustained release of tetracycline over 25 days (cumulative release: ~40–60%). The implants inhibited the growth of *S. aureus* and *E. coli*.	None reported	[58]
Implant	PLA, β-TCP, titanium nitride, doxorubicin	MEX	Mouse	The implants demonstrated an initial burst release phase of doxorubicin for 24 h followed by a gradual release phase over 48 days (cumulative release: ~60%).	Over 18 days, the doxorubicin-loaded implants significantly reduced the growth of subcutaneous K7M2-WT tumors in mice.	[59]
Ocular insert	PLA, glycosomes, ganciclovir	MEX	Rabbit	The glycerosomes demonstrated a sustained release of ganciclovir over 24 h in vitro.	There was a sustained release of ganciclovir over 5 days. The device did not cause gross or histological abnormalities.	[50]
Tablet	PLA, alginate, 5-fluorouracil	MEX	In vitro	The tablets demonstrated an initial burst release phase of 5-fluorouracil for the first 2 h at pH 7.4 (~40%) followed by a gradual release phase up to 9 h (cumulative release: 80–100%).	None reported	[52]
Tablet	PLA, PVA, domperidone	MEX	Rabbit	The tablet demonstrated an initial burst release phase of domperidone for the first 4 h (~65%) followed by a gradual release phase over 10 h (cumulative release: 98%).	The tablet floated in the rabbit’s stomach for at least 10 h.	[53]
Tablet	PLA, riboflavin	MEX	Rabbit	The tablets demonstrated a cumulative release of riboflavin of 41–62% over 72 h.	The tablet floated in the rabbit’s stomach for over 72 h.	[54]
Vaginal rings	PLA, PCL, PEG, progesterone	MEX	In vitro	The rings showed an initial burst release phase over 24 h followed by a gradual release phase over 7 days at a rate of 100–200 μg per day.	None reported	[51]
Vascular graft	PLA, PCL, PEG, NO	MEX	Chick embryo	The graft demonstrated an initial burst release during the first day followed by a gradual release of NO in the physiological range over 14 days.	The NO-loaded graft significantly increased the number of vascular junctions in the chorioallantoic membrane of the chick embryo.	[33]

Abbreviations: β-TCP, beta-tricalcium phosphate; MEX, material extrusion; NO, nitric oxide; PCL, poly(caprolactone); PLA, poly(lactic acid); PEG, poly(ethylene glycol); PVA, poly(vinyl alcohol); VP, vat photopolymerization.

## 5. Medical Devices, Prosthetics, and Orthotics

Three-dimensional-printed PLA has garnered considerable attention in the development of medical devices, prostheses, and orthoses due to its biocompatibility, adaptability, and affordability. The FDA defines a medical device as an instrument, apparatus, implement, machine, contrivance, implant, in vitro reagent, or other related article intended for use in the diagnosis, cure, mitigation, treatment, or prevention of disease in man or other animals [60,61]. PLA’s ability to be easily molded into complex shapes makes it ideal for fabricating different types of medical devices, such as detection electrodes, personal protective equipment, probes, stents, and swabs (Table 4). Three-dimensional-printed PLA is widely used in the medical device area for creating detection electrodes with a good limit of detection. In this application, PLA is typically mixed with conductive materials, such as carbon black, graphene, or graphite. PLA-based electrodes have been used to detect different substances, such as carbendazim [62], creatinine [63], dopamine [64], hydroxychloroquine [65], paraquat [62], quetiapine [66], and sulfanilamide [67]. Biological molecules, such as Hantavirus Araucaria nucleoprotein [68], SARS-CoV-2 cDNA [69], and SARS-CoV-2 S protein [70], have also been detected using 3D-printed PLA electrodes. Drug-eluting medical devices have been discussed in the previous section, and the specific medical devices that have been developed for surgery and radiotherapy are discussed in the next sections.

**Figure 4:**
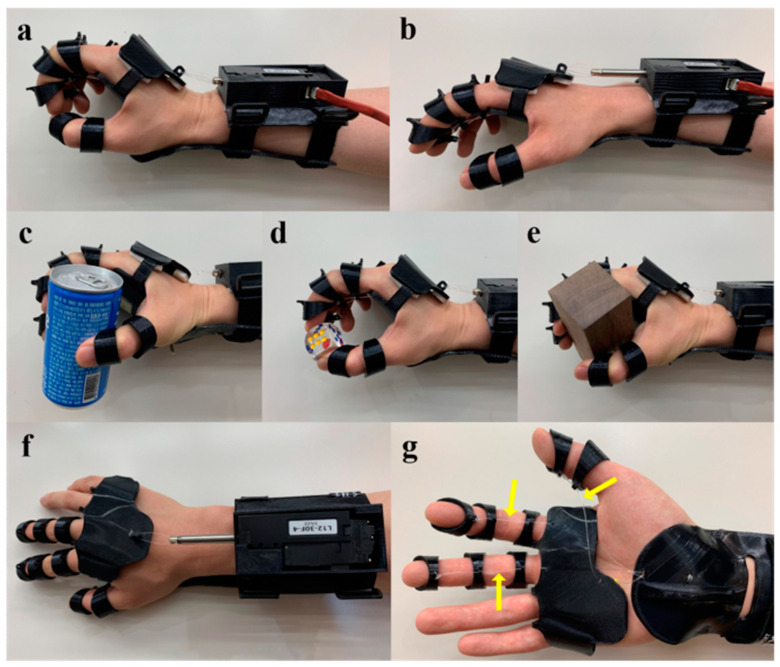
Three-dimensional-printed PLA-based orthotic device. The electromyography-responsive hand orthosis has a linear motor that can be activated to assist in wrist extension and facilitate the tenodesis grasp (**a**). Once the motor goes back into place, the extended wrist relaxes back into a neutral position (**b**). The device has been used to pick up a can, dice, and a wooden block (**c**–**e**). Top view of the device (**f**). Bottom view of the device (**g**) showing the nylon threads (yellow arrow) connecting the splint and rings. Images were adapted with permission from Yoo et al. [82].

Due to its strength and light weight, PLA is suitable for the fabrication of prosthetic and orthotic devices (Table 5, Figure 4), which are a subset of devices that enable people with physical impairments or functional limitations to live healthy, productive, independent, and dignified lives. Prosthetics is the science and art that focus on the development and application of prostheses, which are artificial replacements for absent or deficient bodily components [71]. Three-dimensional-printed PLA has been used to manufacture scleral [72], dental [73,74], hand [75,76], and transtibial prostheses [77]. On the other hand, orthotics is the science and art that focus on the development and application of orthoses, which are externally applied devices used to modify the structural and functional characteristics of the neuromuscular and skeletal systems [71]. Similarly, 3D-printed PLA has been utilized to generate ankle–foot [78], arm [79], and hand orthoses [80]. Exoskeletons are a subset of orthotic devices that differ from conventional orthoses because they utilize an external power source to produce or supplement body movements through components that rely on controllers or sensors. Three-dimensional-printed PLA has been used to produce exoskeletons for the arms [81] and hands [82,83]. 

One benefit of external devices, as opposed to implanted devices, is that they are easier to test in patients. Therefore, several prosthetic and orthotic devices have undergone testing in human subjects. For example, hand orthoses 3D-printed from PLA have been shown to produce patient satisfaction ratings comparable to those of commercially available hand splints [80]. In another study, a dynamic wrist–hand orthosis made from 3D-printed PLA was developed to assist a patient in performing active wrist extension against spastic flexor muscles, demonstrating the adaptability of this technology [84]. Hand exoskeletons made of 3D-printed PLA have also been tested in both healthy subjects and patients with spinal cord injury. A study on healthy subjects demonstrated that exoskeletons can grasp, hold, and release objects with good latency and high accuracy [85]. In another study, the incorporation of haptic stimulations into the exoskeleton enhanced the attention of participants during hand exercises, showcasing the potential for improving the therapeutic outcomes [83]. More importantly, it has been shown that an electromyography-responsive hand exoskeletons can significantly improve the hand function and eating independence in patients with spinal cord injury-induced weakness [82]. With the advancements in 3D printing technology, PLA will continue to play a crucial role in revolutionizing patient care and rehabilitation by offering personalized, high-quality, and affordable solutions in the field of medical devices, prosthetics, and orthotics. Nonetheless, future investigations should explore a wider range of orthotic and prosthetic devices for various indications and involve a larger and more diverse patient population to assess their long-term efficacy and impact on functional independence and the overall rehabilitation goals.

**Table 4:** Recent studies on miscellaneous 3D-printed PLA-containing medical devices.

Device	Device Components	Printing Technique	Results	Reference
Detection electrode	PLA, carbon black	MEX	The electrode showed a linear response for the detection of Hantavirus Araucaria nucleoprotein from 30 to 240 μg/mL in human serum (LOD: 22 μg/mL).	[68]
Detection electrode	PLA, carbon black	MEX	The electrode showed a linear response for the detection of hydroxychloroquine from 0.4 to 7.5 μmol/L (LOD: 0.04 μmol/L).	[65]
Detection electrode	PLA, carbon black	MEX	The electrode showed a linear response for the detection of quetiapine from 5 to 80 × 10^−7^ mol/L (LOD: 2 × 10^−9^ mol/L).	[66]
Detection electrode	PLA, carbon black	MEX	The electrode showed a linear response for the detection of sulfanilamide from 1 to 39.2 μmol/L in breast milk, synthetic urine, and otologic solution samples (LOD: 12 nmol/L).	[67]
Detection electrode	PLA, carbon black	MEX	The electrode showed a linear response for the detection of creatinine from 0.5 to 35 mmol/L (LOD: 37.3 μmol/L).	[63]
Detection electrode	PLA, graphene	MEX	Using different electrochemical techniques, the LOD for dopamine was determined to be around 1.67–2.17 μmol/L.	[64]
Detection electrode	PLA, graphene, gold particles	MEX	The LOD for creatinine and SARS-CoV-2 cDNA were 0.016 mmol/L and 0.3 µmol/L, respectively.	[69]
Detection electrode	PLA, graphite	MEX	The electrode showed linear responses for the detection of paraquat (0.05–1 µmol/L and 1–50 µmol/L) and carbendazim (0.5–50 µmol/L). The LOD for paraquat and carbendazim were 0.01 and 0.03 µmol/L, respectively.	[62]
Detection electrode	PLA, graphite	MEX	The electrode showed a linear response for the detection of SARS-CoV-2 S protein from 5 to 75 nmol/L (LOD: 1.36 nmol/L).	[70]
Face mask	PLA, non-woven fabric	MEX	The mask showed a viral filtration efficiency of at least 80%.	[86]
Face mask extenders	PLA	MEX	The mask extender, which can last for around 2 months, was well-received by healthcare providers.	[87]
Face shield adapter	PLA	MEX	The headlight face shield adapter was used for 2 weeks and has been found to be useful for treating epistaxis, changing tracheostomy cannulas, and routine nasal and oral examinations while protecting the physicians from respiratory droplets, blood, sputum, and other fluids.	[88]
Photothermal probe	PLA, graphene oxide	MEX	The probe demonstrated a photothermal conversion efficiency of up to 32.6%	[89]
Stent	PLA	MEX	The stent exhibited shape recovery following thermomechanical programming.	[90]
Stent	PLA, PU	MEX	The stent with spirals demonstrated self-expansion, anti-migration, and non-cytotoxicity.	[91]
Swab	PLA	MEX	The printed swabs demonstrated threshold cycle values comparable to control swabs in terms of detecting RNase P expression.	[92]
Swab	PLA	MEX	The overall concordance between the prototype and control swabs was 80.8%.	[93]

Abbreviations: LOD, limit of detection; MEX, material extrusion; PLA, poly(lactic acid); PU, poly(urethane).

**Table 5:** Recent studies on 3D-printed PLA-containing prosthetics, orthotics, and sensors.

Device	Device Components	Printing Technique	Results	Reference
Ankle-foot orthosis	PLA, carbon fiber	MEX	The addition of carbon black increased the stiffness and mechanical strength of the device. The PLA and PLA-carbon black devices fractured at loads of 286.2 N and 345.4 N, respectively.	[78]
Arm exoskeleton	PLA	MEX	The exoskeleton can move the arm into eight different positions.	[81]
Arm orthosis	PLA	MEX	The use of perforations reduced the weight of the splints. In comparison to the solid splint, the topology-optimized and square-perforated hand splints were 26% and 12% lighter, respectively.	[79]
Arm sensor	PLA, PU	MEX	The wearable forearm band allowed the acquisition of surface EMG signals for six different movements from 15 volunteers. The classification algorithm for the detected signals had an accuracy of 85%.	[94]
Dental prosthesis	PLA	MEX	The deviation of PLA and wax dentures from the plaster model were comparable.	[73]
Dental prosthesis	PLA	MEX	The temporary crowns were maintained in five patients without fracture, dislodging, or discomfort until the permanent prostheses were ready.	[74]
General sensor	PLA	MEX	The device demonstrated a power density of ~25 mW/m2 and an ability to record the bone–joint motion, coughing action, and foot pressure distribution.	[95]
Hand exoskeleton	PLA	MEX	The EMG-responsive device significantly improved the hand function and eating independence of patients with weakness due to spinal cord injury.	[82]
Hand exoskeleton	PLA	MEX, MJT	The addition of haptic stimulations to the hand exoskeleton improved the attention of the participants during the conducting of hand exercises.	[83]
Hand orthosis	PLA	MEX	The satisfaction ratings for the 3D-printed and commercially available splints were comparable among patients with an indication for immobilization of at least 4 weeks.	[80]
Hand prosthesis	PLA, PU, Nylon	MEX	The body-powered prosthetic hand can perform different grasping patterns due to the adaptivity provided by the articulated fingers.	[75]
Hand prosthesis	PLA	MEX	The prosthetic hand can perform fine movements and grasp different objects.	[76]
Scleral prosthesis	PLA	MEX	A scleral cover shell prosthesis was developed from the corneoscleral topography profile of a volunteer patient.	[72]
Transtibial prosthesis	PLA	MEX	The printed transtibial socket passed the ultimate static test force of 4025 N.	[77]
Wrist hand orthosis	PLA	MEX	A dynamic orthosis was produced to help the patient perform an active extension of the wrist against the spastic flexor muscles.	[84]

Abbreviations: EMG, electromyography; MEX, material extrusion; MJT, material jetting; PLA, poly(lactic acid); PU, poly(urethane).

## 6. Surgical Instruments and Guides

**Figure 5:**
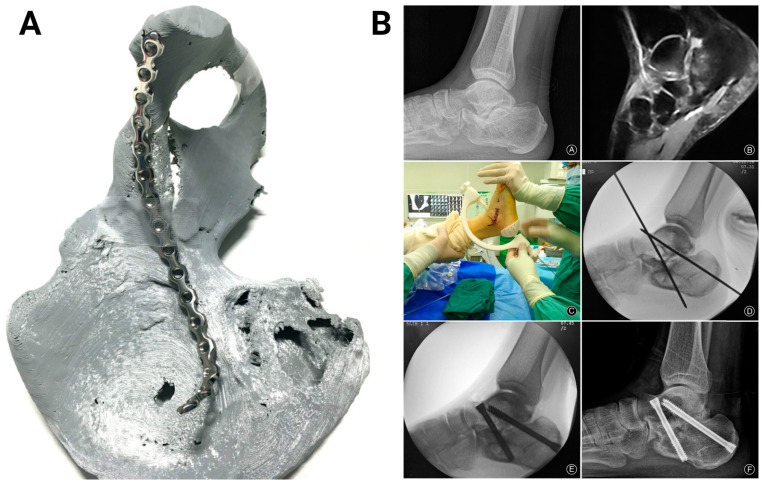
Three-dimensional-printed PLA-based surgical guides. (**A**) The 3D-printed acetabular fracture model served as a guide for pre-bending the steel reconstruction plate prior to surgery. The image was adapted with permission from Weidert et al. [100]. (**B**) A clinical case showing the subtalar joint space upon radiography (top left) and osteoarthritis upon magnetic resonance imaging (top right). The 3D-printed guide was used to position the Kirschner wires (middle). Intraoperative fluoroscopy confirmed the satisfactory placement of the wires (bottom left). After 1 year, radiography showed subtalar bony fusion and obliteration of subtalar joint space (bottom right). Images were adapted with permission from Duan et al. [105].

The rapid prototyping and production of surgical instruments and guides (Table 6, Figure 5) has been made possible via the utilization of 3D printing technology with PLA. PLA’s compatibility with standard sterilization processes makes it suitable for use in sterile surgical environments. Surgical instruments used in septoplasty, such as a scalpel handle, needle holders, toothed forceps, a Cottle/Freer elevator, and a Killian’s speculum, have been produced from 3D-printed PLA [96]. In addition, recent studies have also demonstrated the ability of 3D-printed PLA to generate an external fixator [97], orthopedic screws [98], and a trocar spacer for vitreoretinal surgery [99]. 

The ability of 3D-printed PLA to be precisely engineered into complex shapes has allowed the customization not only of surgical instruments to match specific surgical procedures and patient anatomy but also of surgical guides. A surgical guide is a custom-made model, typically generated from patient-specific medical imaging data, that aids in the precise placement of implants or the execution of surgical procedures with enhanced accuracy and efficiency. It can also minimize the risk of adverse reactions during surgical interventions, contributing to better patient outcomes. Examples of clinical procedures where 3D-printed PLA has been utilized as a guide include acetabular fracture surgery [100], halo pin placement [101], leg osteotomy [102], microtia reconstruction [103], pelvic reconstruction [104], and subtalar joint arthrodesis [105]. For acetabular fracture surgery, the 3D-printed models of the fracture site have been used to preoperatively bend the reconstruction plate in a total of 12 cases [100]. For halo pin placement, PLA models have successfully guided safe placement in two pediatric patients with complex spinal conditions, leading to successful skull fusion [101]. For leg osteotomy, a PLA model was used to verify the cutting angle and positions for a patient with X-linked hypophosphatemia and 35-degree left genu varum [102]. For microtia reconstruction, PLA ear models proved valuable as intraoperative references [103]. Lastly, for subtalar joint arthrodesis, PLA surgical navigation guides reduced the procedure times without affecting the fusion outcomes [105].

While several surgical procedures have seen success using 3D-printed PLA guides in human studies, more complex procedures like cervical screw fixation have only been tested in preclinical models [106,107]. However, these promising preclinical results pave the way for future investigations that could bridge the gap to clinical translation. Further research is crucial to expand the application of PLA surgical guides to a wider range of procedures, including highly complex surgeries like cardiovascular and neurosurgery where precise and personalized instrumentation is paramount. Addressing these challenges and expanding the scope of PLA’s applications will unlock its full potential in revolutionizing surgical practice.

**Table 6:** Recent studies on 3D-printed PLA-based surgical guides.

Procedure	Image Source	Guide Components	Printing Technique	Results	References
Acetabular fracture surgery	CT	PLA	MEX	The surface filtering pipeline decreased the printing time by 65%. The mean average deviation of the printed models from the computed model ranged from 0.67 to 1.06 mm. The mean reduction time of the fracture fragments during the operation was 12 min and 42 s. After 12 months, the average Harris Hip Score, Modified Harris Hip Score, and Merle d’Aubigne Score of nine patients were 71.6 points (fair), 75.7 points (fair), and 11.1 points (poor), respectively.	[100]
Ankle, foot, and pelvic reconstruction	CT	PLA	MEX	The reconstruction of the deformities was planned using the printed models.	[108]
Cervical screw fixation	CT	PLA	MEX	Patient-specific models and drill guides were fabricated for simulating C1/2 cervical pedicle screw fixation. The drill guides had an accuracy of 93.54%.	[106]
Cervical screw fixation	CT	PLA	MEX	Patient-specific navigation templates were fabricated for lower cervical anterior transpedicular screw insertion. The axial and sagittal accuracies were 99.5% and 97%, respectively.	[107]
Dental implant surgery	Surface scanning	PLA, PHA	MEX, MJT	The printed materials showed significant angle deviation and apex offset after sterilization. However, the errors were comparable to the levels reached using the materials that are routinely used in clinical settings.	[109]
Halo pin placement	CT	PLA	MEX	The skull models were used to guide safe pin placement in two pediatric patients with diastrophic dysplasia requiring prolonged pre-fusion halo-gravity traction for staged fusion for progressive kyphoscoliosis. Both patients achieved fusion union by 9 months.	[101]
Leg osteotomy	CT	PLA	MEX	The model was used to verify the cutting angle and positions of the osteotomy for a patient with X-linked hypophosphatemia and 35-degree left genu varum. After this, an arch osteotomy of the left femur and transverse osteotomy of the left upper tibiofibular bone were performed. The genu varum was repaired and the leg was fixed using an Ilizarov external fixator.	[102]
Microtia reconstruction	Surface scanning	PLA	MEX	A patient-specific ear model was developed without additional ionizing radiation exposure and served as a helpful intraoperative reference for microtia reconstruction.	[103]
Pelvic reconstruction	CT	PLA, titanium alloy	MEX	Compared to the common 3D-printed anatomic template, the modified 3D-printed anatomic template with a customized cutting block resulted in a shorter operating time (209 vs. 272 min) and less blood loss (1390 vs. 2248 mL) in patients requiring pelvic reconstruction after pelvic tumor resection. The modified strategy also reduced the local tumor recurrence (5.26 vs. 42.11%), but it is associated with a higher rate of implant loosening (21.05 vs 0%).	[104]
Subtalar joint arthrodesis	CT	PLA	MEX	The surgical guide reduced the time that it took to drill the Kirschner wire to a satisfactory position in patients who had undergone subtalar joint arthrodesis compared to the control group (2.1 vs. 4.6 min). After 1 year, there was no significant difference observed in the subtalar fusion time and American Orthopaedic Foot & Ankle Society scores between the two groups.	[105]

Abbreviations: CT, computed tomography; EMG, electromyography; MEX, material extrusion; MJT, material jetting; PHU, poly(hydroxyalkanoate); PLA, poly(lactic acid).

## 7. Radiotherapy Devices and Phantoms

**Figure 6:**
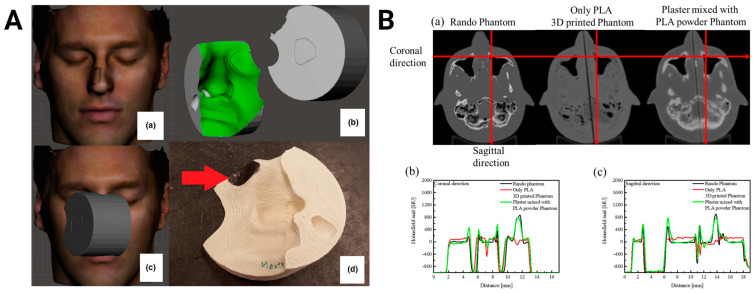
Three-dimensional-printed PLA-based radiotherapy devices. (**A**) The process to produce a bolus includes an optical scan of the patient (a), a digital design of the material (b), optimization of treatment geometry (c), and printing and placing lead shielding as indicated by the red arrow (d). Images were adapted with permission from Sasaki et al. [120]. (**B**) Computed tomography scans of a commercial Rando head phantom, a head phantom printed using only PLA, and a head phantom printed with a mixture of plaster and PLA powder (a). Radiopacity profiles along the red line in the transverse (a) and coronal (b) planes. Images were adapted with permission from Kim et al. [113].

Radiotherapy is a frequently used cancer treatment modality, and it involves the use of ionizing radiation to control or eradicate cancer cells. Three-dimensional printing has allowed the development of customized radiotherapy devices and phantoms that can help enhance the precision of treatment delivery while minimizing the exposure of healthy tissues to radiation (Table 7, Figure 6). Examples of radiotherapy devices that have been produced from 3D-printed PLA include a beam modifier for total skin electron beam treatment [110], a bolus for postmastectomy radiation [111], and an applicator for gynecologic brachytherapy [112]. Additionally, PLA has been combined with different polymers and radiopaque materials to construct 3D-printed phantoms for quality assurance and dosimetry calibration in the context of treatment planning and verification. Recent studies have reported the development of 3D-printed phantoms of the head [113], cervical spine [114], breast [111,115,116], thorax [117,118], and pelvic organs [119]. 

The clinical impact of 3D-printed PLA in radiotherapy has been demonstrated in two recent studies. Digitally designed PLA boluses have been successfully used in nine patients with a complex surface anatomy for radiation, highlighting the clinical feasibility of personalized 3D-printed devices [120]. In another study, the use of PLA templates for radioactive seed implantation in lung cancer patients led to significantly improved dosimetric coverage of the tumor compared to the control group [121]. Building on these successes, ongoing innovations in composite materials can facilitate the development of even more personalized radiotherapy devices and complex phantoms. These advancements are poised to enhance the treatment precision and accuracy by closely mimicking the heterogeneity of the human anatomy, leading to more accurate dosimetry and treatment planning.

**Table 7:** Recent studies on 3D-printed PLA-containing radiotherapy devices and phantoms.

Device	Image Source	Device Components	Printing Technique	Results	Reference
Beam modifier	N/A	PLA	MEX	The printed modifier shaped the electron beam as desired, resulting in an adequate field and skin coverage for total skin electron beam treatment with 10% and 3% inhomogeneity in the vertical and lateral dimensions, respectively.	[110]
Bolus	CT	PLA	MEX	The printed bolus for postmastectomy radiation had a significantly lower frequency of air gaps of at least 5 mm in length compared to a standard sheet bolus (13% vs. 30%). The surface dose was within 3% for both bolus types.	[111]
Bolus	CT, surface scanning	PLA	MEX	The digitally designed boluses had an average shape error of less than 0.5 mm and were used for the treatment of nine patients with a complex surface anatomy with no issues.	[120]
Brachytherapy applicator	CT	PLA, tungsten	MEX	The patient-specific applicators can provide comparable doses while offering advanced healthy tissue sparing. The PLA-tungsten composite acted as the shielding material.	[112]
Dosimetry guide	CT	PLA	MEX	The group of lung cancer patients with the printed template for the implantation of 125I radioactive seeds had a greater mean postoperative V90 value compared to the control group (93.8 vs. 88.42%).	[121]
Phantom	CT	PLA	MEX	Compared to the computational phantom, the skin, adipose, glandular, and tumor tissues in the printed breast phantom had a percentage difference of 1.8%, 10.1%, 4.5%, and 12.3%, respectively.	[122]
Phantom	CT	PLA, ABS	MEX	PLA (188 HU) and ABS (24 HU) were used to mimic glandular and adipose tissues in the breast, respectively.	[115]
Phantom	CT	PLA, ABS, iron	MEX	The phantom slab demonstrated a similar range of HU that is comparable to the commercial phantom. The mean HU values for lung tissue, soft tissue, low-density bone, and high-density bone were –760, 50, 220, and 630, respectively.	[117]
Phantom	CT	PLA, bismuth oxide	MEX	The addition of bismuth oxide significantly enhanced the radiopacity of the cervical spine phantom compared to plain PLA (612 HU vs. 119 HU).	[114]
Phantom	CT	PLA, iron	MEX	The pediatric torso phantom demonstrated an excellent similarity to commercially available phantoms. The mean HU values for the heart, soft tissue remainder, right lung, and vertebral body were 94, 31, −417, and 1180, respectively.	[118]
Phantom	CT	PLA, plaster	MEX	The head and neck phantom showed a mean difference of 61 HU for soft tissue and 109 HU for bone tissue compared to the Rando phantom.	[113]
Phantom	CT	PLA, PP	MEX	The Pearson’s coefficients between the matched CT images of the breast phantom with those of the patients were 0.91–0.97.	[116]
Phantom	CT	PLA, StoneFil	MEX	The HU values for the hip bone phantoms varied between 700 and 800.	[123]
Phantom	N/A	PLA, PVC, silicone	MEX	PLA and heat-resistant silicone were used to fabricate the phantom case, prostate casting molds, probe insert, and prostate positioning tool, while PVC was used to fabricate the prostatic tissue.	[119]
Phantom	N/A	PLA, zirconium oxide	MEX	The composites with a mere 6% weight fraction demonstrated a maximal radiopacity of 184 HU.	[124]

Abbreviations: ABS, acrylonitrile butadiene styrene; CT, computed tomography; HU, Hounsfield unit; MEX, material extrusion; PLA, poly(lactic acid); PP, poly(propylene); PVC, poly(vinyl chloride).

## 8. Training Models and Simulators

**Figure 7:**
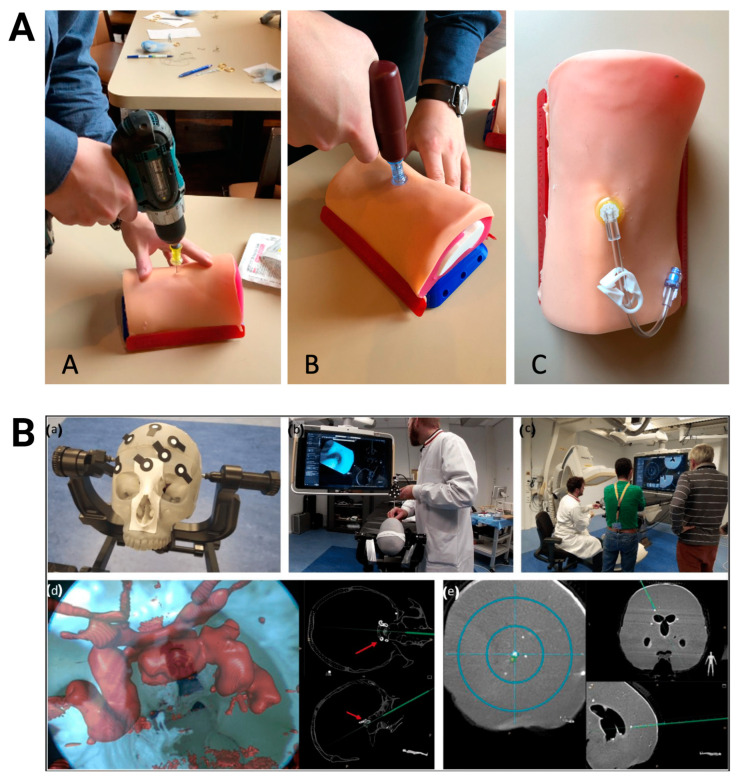
Three-dimensional-printed PLA-based training models. (**A**) Testing of adult proximal tibia intraosseous trainer with a cordless drill (A), EZ-IO power driver (B), and needle insertion (C). Images were adapted with permission from Engelbrecht et al. [133]. (**B**) Anthropomorphic skull and brain model blocked on a clamp (a) for use in augmented reality-navigated endonasal skull-base surgery simulation (b/d) and brain biopsy simulations (c/e). Images were adapted with permission from Lai et al. [147].

Three-dimensional-printed PLA has also played a pivotal role in the development of training models and simulators (Table 8, Figure 7). The versatility and affordability of PLA have enabled the creation of anatomically accurate models and simulators that reflect real-world clinical scenarios, allowing medical professionals and trainees to practice procedures and techniques in a controlled environment. PLA-based simulators have been developed to train medical personnel in a wide variety of procedures, such as bronchoscopy [125], cervical myelography [126], craniosynostosis correction [127], dental implant surgery [128], digital rectal examination [129], endoscopic third ventriculostomy [130], external ventricular drain placement [131], intraarticular joint injection [132], intraosseous access [133,134], mastoidectomy [135,136], maxillofacial surgery [137], nasal osteotomy [138], neuraxial anesthesia delivery [139], pedicle screw insertion [140], and transcranial ultrasonography [141]. 

The use of PLA-based constructs offers a cost-effective, accessible, and safer means of enhancing medical training and skill development, which could ultimately improve patient care and safety. To fully unravel its potential in medical training and education, several key areas require further development. Optimizing the printing processes and material usage can enhance the cost-effectiveness and accessibility, making this technology more widely available, particularly in resource-limited settings. Additionally, establishing standardized evaluation protocols and validating the effectiveness of models and simulators across various medical specialties and training programs are crucial for broader adoption. Lastly, integrating 3D-printed models with cutting-edge technologies like virtual reality can create more immersive and interactive training environments that enhance learning experiences and improve skill acquisition. Overall, these strategies could revolutionize medical education by providing more realistic simulations, ultimately leading to well-trained healthcare providers and improved patient outcomes.

**Table 8:** Recent studies on 3D-printed PLA-containing training models and simulators.

Device	Image Source	Device Components	Printing Technique	Results	References
Aneurysm model	MRA	PLA	MEX	CT measurements of the intracranial aneurysm models were 0.32–0.35 mm higher than the MRA measurements.	[142]
Bone model	CT	PLA	MEX	The ultimate strength of the printed bones (3920–8677 N) was closer to that of real bones (4992–13620 N) than the commercially available polyurethane-based products (1000–9119 N).	[143]
Bone model	CT	PLA	MEX	The participants were 130 third-year medical students. The group with the printed craniofacial bone models had a higher gain score in anatomical knowledge than the cadaveric skull group (50 vs. 37.3).	[144]
Bone model	CT	PLA	PBF	The correlation coefficient of the mechanical properties between the printed and actual trabeculae reached up to 0.94.	[145]
Bone model	N/A	PLA, PVA	MEX	The vertebral models were successfully instrumented without hardware or material failures.	[146]
Brain model	CT, MRI	PLA, PVA, calcium carbonate	MEX	The skull was printed using PLA with calcium carbonate. The brain was cast using a mixture of water, coolant, PVA, and barium sulfate.	[147]
Brain model	MRI	PLA, PAM	MEX	The brain phantom was fabricated by extruding PAM into a PLA skull.	[148]
Bronchoscopy simulator	CT	PLA, PVA, silicone	MEX	All the 17 participants had performed >100 bronchoscopies in the past. Of these, 77% thought that the printed stenotic airway model was better or much better for airway inspection when compared with the Broncho-Boy. A total of 94% reported that the model was accurate or very accurate for realism.	[125]
Cervical myelography simulation	CT	PLA	MEX	The simulator adequately demonstrated the dimensions of the cervical canal in static and dynamic positions.	[126]
Congenital heart disease model	CTA	PLA	MEX	The CT imaging of the models matched the original digital models. Independent reviewers correctly described 80 and 87% of the congenital heart disease models, respectively.	[149]
Craniosynostosis correction simulator	CT	PLA	MEX	The participants were five attending physicians, four fellows, and nine residents. Of the participants, 100% and 94% reported that the model was a valuable training tool for open reconstruction and endoscopic suturectomy, respectively.	[127]
Dental implant surgery simulator	CT	PLA	MEX, MJT, PBF, VP	The MJT model had the highest scores from five maxillofacial surgeons for drilling perception and corticotrabecular transition.	[128]
Digital rectal examination simulator	N/A	PLA, silicone	MEX	The participants were five urologists. The participants gave a rating of 4.24 out of 5 to the task trainer with regard to appropriateness and usefulness in education.	[129]
Endoscopic third ventriculostomy simulator	CT, MRI	PLA, silicone	VP	The participants were three attending physicians and 12 residents. A total of 87% strongly agreed that the simulator was useful for resident training, while 93% strongly agreed that the simulator helped them understand how to orient themselves with the endoscope.	[130]
External ventricular drain placement simulator	N/A	PLA, agar	MEX	The participants were five neurosurgery residents and six EM residents. One hundred percent strongly agreed that the model was useful for their training and was a realistic simulation of the procedure.	[131]
Intraarticular joint injection simulator	N/A	PLA, ballistics gel	MEX	The model reproduced the finding of an anterior shoulder dislocation upon ultrasound imaging and allowed for the visualization of the needle within the joint space.	[132]
Intraosseous access simulator	N/A	PLA, silicone	MEX	The participants were 15 rural family medicine residents, six rural EM physicians, and six registered nurses. The majority of the physicians considered the adult intraosseus access simulator to be very effective as a training tool.	[133]
Intraosseous access simulator	N/A	PLA, silicone	MEX	The participants were seven EM physicians, two family medicine residents, and three medical students. The majority of the physicians found the pediatric intraosseous access simulator to be “very effective”, whereas the medical students found it to be “effective to very effective”.	[134]
Mastoidectomy simulator	CT	PLA	MEX	The participants were 10 junior residents. The ratings for the ease of use, safety, and value in training were 4.75/5, 4.5/5, and 4.35/5, respectively.	[135]
Mastoidectomy simulator	CT	PLA, PETG, ABS, PC, nylon	MEX	The participants were surgeons with an average of 56.5 temporal bone procedures. PETG had the highest score for haptic feedback and appearance, which were 8.3/10 and 7.6/10, respectively. PLA was a reliable alternative with scores of 7.4/10 and 7.6/10.	[136]
Maxillofacial surgery simulation	CT	PLA, ABS, silicone	MEX	The participants were 10 maxillofacial surgeons. The majority rated the haptics of bone and soft tissue simulation “good” and “moderate to good”, respectively.	[137]
Nasal osteotomy simulator	CT	PLA	MEX	The evaluators were two attending facial plastic surgeons and one facial plastic surgery fellow. All of them strongly agreed that the model with a 10% infill density mimicked human bone better than the other models with different infill densities.	[138]
Neuraxial anesthesia simulator	CT	PLA, gelatin, psyllium fiber	MEX	The participants were 22 anesthesiologists. Although the model was found to be less realistic in terms of surface palpation than the Simulab phantom, it had significantly better fidelity for the loss of resistance, dural puncture, and ultrasound imaging.	[139]
Pedicle screw insertion simulator	CT	PLA, ABS, nylon	MEX	The surgeon authors reported that the ABS models were much more similar to human bone than the PLA models when cannulating pedicles and placing screws.	[140]
Transcranial ultrasonography simulator	CT, MRI	PLA, PVC, photopolymer resin	MEX, VP	The transcranial ultrasonography of the phantom proved the possibility of B-mode imaging differentiation between the brain and particles of metal, bone tissue, and PLA. However, when examined through the temporal bone model, the particles in the sonogram were 3–10 times larger than their actual size.	[141]

Abbreviations: ABS, acrylonitrile butadiene styrene; CT, computed tomography; CTA, CT angiography; EM, emergency medicine; MEX, material extrusion; MJT, material jetting; MRI, magnetic resonance imaging; MRA, MR angiography; PAM, poly(acrylamide); PBF, powder bed fusion; PC, poly(carbonate); PETG, poly(ethylene terephthalate glycol); PLA, poly(lactic acid); PVA, poly(vinyl alcohol); PVC, poly(vinyl chloride); VP, vat photopolymerization.

## 9. Conclusions

In summary, PLA’s adaptability, biocompatibility, and cost-effectiveness have made it an indispensable tool in addressing a wide range of medical challenges, from tissue engineering to medical education and training. While the progress is remarkable, further research is imperative to maximize the utility of 3D-printed PLA in medicine. In tissue engineering, reproducing the intricate architecture, hierarchy, and heterogeneity of native bone tissue remains a significant challenge. However, emerging high-resolution printing methods and strategies to enhance the vascularization offer promising solutions. In drug delivery, optimizing the scaffold and device composition to ensure the stability, safety, and efficacy of the incorporated drugs should be a key focus of further research. For medical devices, expanding the range of the 3D-printed PLA prosthetics and orthotics for diverse indications and patient populations is crucial to realizing their full potential. In the context of surgery, further research is needed to extend the application of PLA surgical guides to highly complex procedures where precision and personalization are of paramount importance. In the area of radiotherapy, the continued innovation in composite materials should facilitate the development of more personalized devices and phantoms that closely mimic the heterogeneity of the human anatomy, ultimately leading to more accurate dosimetry and treatment planning. Finally, in medical training and education, optimizing the printing processes, establishing standardized evaluation protocols, and integrating with virtual learning strategies, are key steps toward broader and more meaningful adoption. With these research opportunities, the 3D-printed PLA-based materials are expected to continue inspiring innovation, transforming patient care, and shaping the future of healthcare delivery and personalized medicine.
